# Emergency Portacaval Shunts: Is Orloff Correct?

**DOI:** 10.1155/1997/80586

**Published:** 1997

**Authors:** Stuart J. Knechtle, Layton F. Rikkers

**Affiliations:** Department of Surgery University of Wisconsin Hospital and Clinic H4/710 Clinical Science Center 600 Highland Avenue Madison WI 53792 USA

## Abstract

A prospective randomized trial was conducted in unselected, consecutive pateints with bleeding esophageal varices resulting from cirrhosis comparing (1) emergency portacaval shunt performed within 8 hr of initial contact (21 patients) with (2) emergency medical therapy (intravenous vasopressin and esophageal balloon tamonade) followed in 9 to 30 days by elective portacaval shunt in survivors (22 patients). All patients underwent the same diagnostic workup within 3 to 6 hr of initial contact, and received indentical supportive therapy initially. All patients were followed up for atleast 10 hr. The protocol contained no escape or crossover provisions. There were no statistically significant differences between the two treatment groups in the incidence of any of the clinical variables, results of laboratory tests or degree of portal hypertension. Child's risk classes in the shunt group were A-2 patients, B-8 patients and C-11 patients, whereas in the medical group they were A-10 patients, B-5 patients, and C-7 patients, a significant difference (p<0.01) that might have favored emergency medical treatment. Bleeding was controlled initially and permanently by emergency shunt in every patient, but by medical therapy in only 45% (p<0.001). Mean requirement for blood transfusion was 7.1±2.6 units in the shunt group and 21.4±2.6 units in the medical group (p<0.001). Eighty-one percent of the pateints in the shunt group were discharged alive compared with 45% in the medical group (p=0.027). Five- and 10-yr observed survival rates were 67% and 57%, respectively, after emergency shunt compared with 18% and 18%, respectively, after the combination of emergency medical therapy and elective shunt (p<0.01). These survival rates produced by emergency shunt performed within 8 hr of initial contact confirm the effectiveness of this procedure observed in our previous unrandomized studies.


*HPB Surgery*, 1997, Vol. 10, pp. 253–268
Reprints available directly from the publisher
Photocopying permitted by license only

© 1997 OPA (Overseas Publishers Association)
Amsterdam B.V. Published in The Netherlands
by Harwood Academic Publishers
Printed in India

HPB INTERNATIONAL

EDITORIAL & ABSTRACTING SERVICE

JOHN TERBLANCHE EDITOR
Department of Surgery,
Medical School · Observatory 7925
Cape Town · South Africa
Telephone: 27-21-406-6204
Telefax 27-21-448-6461
E-mail: JTER BLAN@UCT GSHI.UCT.AC.2A

### Emergency Portacaval Shunts: Is Orloff Correct?

### ABSTRACT

*Orloff, M.J., Bell, Jr., R. H., Orlo, M.S., Hardison, W.G.M. and Greenburg, A.G. (1994) Prospective randomized trial of emergency portacaval shunt and emergency medical therapy in unselected cirrhotic patients with bleeding varices. Hepatology; 20: 863–872.*

**A prospective randomized trial was conducted in unselected, consecutive pateints with bleeding esophageal varices resulting from cirrhosis comparing (1) emergency portacaval shunt performed within 8 hr of initial contact (21 patients) with (2) emergency medical therapy (intravenous vasopressin and esophageal balloon tamonade) followed in 9 to 30 days by elective portacaval shunt in survivors (22 patients). All patients underwent the same diagnostic workup within 3 to 6 hr of initial contact, and received indentical supportive therapy initially. All patients were followed up for atleast 10 hr. The protocol contained no escape or crossover provisions. There were no statistically significant differences between the two treatment groups in the incidence of any of the clinical variables, results of laboratory tests or degree of portal hypertension. Child's risk classes in the shunt group were A-2 patients, B-8 patients and C-11 patients, whereas in the medical group they were A-10 patients, B-5 patients, and C-7 patients, a significant difference ($p<0.01$) that might have favored emergency medical treatment. Bleeding was controlled initially and permanently by emergency shunt in every patient, but by medical therapy in only 45% ($p<0.001$). Mean requirement for blood transfusion was $7.1 \pm 2.6$ units in the shunt group and $21.4 \pm 2.6$ units in the medical group ($p<0.001$). Eighty-one percent of the pateints in the shunt group were discharged alive compared with 45% in the medical group ($p=0.027$). Five- and 10-yr observed survival rates were 67% and 57%, respectively, after emergency shunt compared with 18% and 18%, respectively, after the combination of emergency medical therapy and elective shunt ($p<0.01$). These survival rates produced by emergency shunt performed within 8 hr of initial contact confirm the effectiveness of this procedure observed in our previous unrandomized studies. (Hepatology 1994; 20: 863–872.)**

*Keywords*: Portacaval shunt, bleeding varices, oesophageal varices portal hypertension

### PAPER DISCUSSION

The study by Orloff and associates on the use of emergency portacaval shunt (EPCS) versus the combination of emergency medical therapy (EMT) and a subsequent elective shunt, suggests a significant benefit to immediate portacaval shunting for patients with bleeding esophageal varices and cirrhosis. Both early and long-term survival rates were significantly greater in the EPCS group. Bleeding was controlled in only 45% of patients treated with EMT, consisting of intravenous vasopressin and esophageal balloon tamponade followed 9 to 30 days later by elective portacaval shunt; the remaining patients in this group died from uncontrolled bleeding during the initial hospitalization. In contrast, bleeding was controlled in 100% of patients undergoing EPCS, 81% of whom were discharged alive. Although long-term differences in survival between the treatment groups are just as impressive as the early results, multiple factors other than the initial treatment selected are likely to have influenced outcomes. The only important end points that should be considered in this trial are control of bleeding and survival during the initial hospitalization.

The authors are to be commended for achieving results with EPCS which are superior to those obtained in another recent controlled trial and in virtually all other uncontrolled series of EPCS[1]. An early mortality rate of only 19% for unselected cirrhotic patients (over 50% of whom were in Child's class C) with acute variceal hemorrhage is impressive indeed. How are Orloff and his associates able to salvage such a high percentage of desperately ill patients when so many others have been unable to even approach these enviable results? Dr. Orloff's contention would be that his approach is unique. No one else has attempted to uniformly accomplish surgical portal decompression within 8 hours of admission for all patients who bleed from varices. He maintains that such early definitive treatment prevents the hepatic functional deterioration that so often plagues the hospital courses of patients who persistently bleed or rebleed. However, in order for such a therapeutic protocol to be effective, early referral to an institution with 24-hour availability of a surgical team with skills and experience commensurate with that of Dr. Orloff's team is essential. Such resources are not available in most areas of the United States and other countries, especially those regions with a large rural population, where patients typically make stops at one or more hospitals before arriving at an institution capable of successfully providing definitive treatment for variceal hemorrhage. Along the way, persistent bleeding frequently leads to deterioration of hepatic function and a risky situation for emergency surgery. Such patients would benefit from initial non-operative therapy if it were reasonably effective. Ideally, such therapy would be widely available including relatively small community hospitals where these patients often first seek medical care.

Another notable finding of this trial, equal in importance to the observation that EPCS salvaged a high percentage of patients, is the general ineffectiveness of non-operative therapy compared to other reports[2,3,4]. Only 36% of bleeding episodes were controlled with the combination of vasopressin and balloon tamponade. The dismal results in the EMT group with respect to bleeding control and early survival likely relate to two factors: 1) ineffectiveness of the medical treatment options used (vasopressin and balloon tamponade), and 2) disallowal of cross-over from the EMT to EPCS group when EMT failed. Our prinicipal concern with this study relates to its relevance to current practice, because endoscopic therapy, which has been demonstrated in multiple controlled trials to be superior to vasopressin infusion and balloon tamponade, was not utilized in the EMT limb.

Although not widely availble when this trial was initiated in 1978, endoscopic treatment (sclerosis and banding) has become the mainstay in most centers for management of the acute variceal bleeder with bleeding control rates in excess of 70% in nearly all series[4,5,6]. Additionally, other effective non-operative therapies such as transjugular intrahepatic portosystemic shunt (TIPS)[7] and intravenous octreotide[4,8] have been introduced since the completion of this trial. Since a TIPS accomplishes portal decompression in a manner similar to a surgical shunt, it would be expected to relieve variceal bleeding as well, with potentially less morbidity, especially in high risk patients. Patients treated with TIPS often improve their liver function, but those who deteriorate due to inadequate functional hepatic reserve can be considered for liver transplantation. Unlike patients with surgical shunts, TIPS does not increase, and in fact may decrease the risk of liver transplantation.

Since the results of this trial, which enrolled patients from 1978 to 1983 but which was not published until 1994, may have limited relevance to the management of acute variceal bleeding in 1996, the results of an ongoing trial of EPCS versus endoscopic treatment by Orloff's group are eagerly awaited. Will this provide a definitive answer? Probably not, because TIPS is now available for high-risk patients who fail emergency endoscopic treatment. In fact, it is unlikely that any single therapy will ever be uniformly applicable to this heterogeneous group of patients. Now that several effective modalities are available, a thoughtful, individualized approach is essential to obtain optimal results.

### *References*

[1] Cello, J.P., Grendell, J.H., Crass, R.A., Weber, T.E., and Trunkey, D.D. (1987). Endoscopic sclerotherapy versus portacaval shunt in patients with severe cirrhosis and acute variceal hemorrhage. *N. Engl. J. Med.*, **316**, 11–15.

[2] Paquet, K.J. and Feussner, H. (1985). Endoscopic sclerosis and esophageal balloon tamponade in acute hemorrhage from esophagogastric varices: a prospective controlled randomized trial. *Hepatology*, **5**, 580–583

[3] Holman, J.M. and Rikkers, L.F. (1980). Success of medical and surgical management of acute variceal hemorrhage. *Am. J. Surg.*, **140**, 816–820.

[4] D'Amico, G., Pagliaro, L. and Bosch, J. (1995). The treatment of portal hypertension: meta-analytic review. *Hepatology*, **22**, 332–354.

[5] Westaby, D. and Williams, R. (1990). Status of sclerotherapy for variceal hemorrhage in 1990. *Am. J. Surg.*, **160**, 32–36.

[6] Stiegmann, G.V., Go, J.S., Michaletz-Onody, P.A., Korula, J., Lieberman, D. and Saeed, Z.A., *et al.* (1992). Endoscopic sclerotherapy as compared with endoscopic ligation for bleeding esophageal varices. *N. Engl. J. Med.*, **326**, 1527–1532.

[7] Ring, E.J., Lake, J.R. Roberts, J.P., Gordon, R.L., LaBerge, J.M. and Read, A.E., *et al.* (1992). Using transjugular intrahepatic portosytemic shunt to control variceal bleeding before liver transplantation. *Ann Intern. Med.*, **116**, 304–309.

[8] Besson, I., Ingrand, P., Person, B., Boutroux, D. and Bernard, P., *et al.* (1995). Sclerotherapy with or without octreotide for acute variceal bleeding. *N. Engl. J. Med.*, **333**, 555–60.

Stuart J. Knechtle, M.D. and
Layton F. Rikkers, M.D.
Department of Surgery
University of Wisconsin Hospital and Clinic
H4/710 Clinical Science Center
600 Highland Avenue
Madison, WI 53792
Tel: (608) 263-1377
Fax: (608) 265-5963

### Prediction of the First Variceal Haemorrhage

### ABSTRACT

*Siringo, S., Bolondi, L., Gaiani, S., Sofia, S., Zironi, G., Rigamonti, A., Di Febo, G., Miglioli, M., Cavalli, G. and Barbara, L. (1994). Timing of the first variceal hemorrhage in cirrhotic patients: Prospective evalua-*

*tion of doppler flowmetry, endoscopy and clinical parameters. Hepatology;* **20**: *66–73.*

**We followed 87 cirrhotic patients with esophageal varices and without previous hemorrhage for a mean period of 24 mo to prospectively evaluate the occurance of variceal bleeding within (early) or after (late) 6 mo from entry and the contribution of portal Doppler ultrasound parameters to the prediction of early and late hemorrhage. Clinical, biochemical, endoscopic and portal Doppler ultrasound parameters were recorded at entry. Variceal bleeding occurred in 22 patients (25.3%). Nine (40.9%) bled within the first 6 mo. Cox regression analysis identified variceal size, cherry-red spots, serum bilirubin and congestion index of the portal vein (the ratio of portal vein [cross-sectional area] and portal blood flow velocity) as the only independent predictors of first variceal hemorrhage. Discriminant analysis was used to find the prognostic index cut off points to identify patients who bled within 6 mo (prognostic group 1) or after 6 mo (prognostic group 2) or remained free of bleeding (prognostic group 3). The cumulative proportion of patients correctly classified was 73% in prognostic group 1, 47% in prognostic group 2 and more than 80% in prognostic group 3. The addition of Doppler ultrasound flowmetry to clinical, biochemical and endoscopic parameter only improved the classification of patients with early bleeding. (Hepatology 1994; 20: 66–73.)**

*Keywords*: Variceal haemorrhage cirrhosis portal hypertension, oesophageal varices

### PAPER DISCUSSION

Approximately, 25 to 30% of cirrhotic patients with esophageal varices and without previous variceal hemorrhage will bleed from ruptured varices and 70% of them will do so within the first two years of follow-up [1]. However, studies evaluating the risk factors for the first variceal bleeding have not assessed the timing of the variceal bleeding during this period [2–4]. The assessment of these patients suggest [5] that the risk of bleeding within the first two years is not constant; it tends to decrease after an inital period of six months. Therefore, the identification of risk factors for early and late bleeding is important because it could allow randomization of patients into different treatment groups in prophylactic trial for the prevention of a first variceal hemorrhage, i.e. by betablockers or endoscopic sclerotherapy.

Risk factors for a high bleeding risk have been described by our group [6], by the Japanese Research Society for Portal Hypertenion[7] and by the North Italian Endoscopy Club[2]. However, in these description the role of portal hemodynamics in the prediction of variceal bleeding are not included. The New Haven Group [8] has described the prevalence of a certain level of portal pressure for the risk of variceal bleeding. Therefore, this additional risk factor was included in a prospective randomized trial of prophylactic endoscopic sclerotherapy of our group and seems to be very useful [9].

However, this selection criterion is invasive and thus has limits. Doppler ultrasound is a new tool for evaluating portal hypertension, providing a non invasive assessment of blood flow in most splanchnic vessels. The current use of doppler ultrasound has focused on studies of pathophysiology of portal hypertention and on assessment of effects of vasoactive drugs on splanchnic blood flow. Early studies of portal hemodynamics in cirrhotic patients may have a potential prognostic value in assessment of the risk of variceal bleeding [12].

Therefore, the group of Siringo prospectively evaluated the (a) occurrence of the first variceal bleeding, defined hemorrhage occurring within six months of entry into control studies, and late first variceal bleeding (i.e. bleeding occuring after six months of follow-up), and (b) the role of portal doppler ultrasound parameters, in addition to clinical and endoscopic criteria, in predicting the early and late occurrence of hemorrhage, as well as the possibility of patients remaining free of bleeding.

Therefore, during the period of 38 months, 95 consecutive cirrhotic patients with esophageal varices and without previous variceal hemorrhage were subjected to doppler ultrasound evaluation. The doppler ultrasound was technical feasible in 87 patients (91,6%), who were subseqently followed up for a mean period of 24,0 (±14,5) months. 71 (81.6%) of the 87 were inpatients when they entered the study. At entry, the patients underwent upper gastrointestinal endoscopy, and varices were classified according to the Japanese Research Society of portal hypertension endoscopic rule [7]. Clinical and biochemical data were recorded in each patient, and the severity of the liver disease was assessed using the Cambell numeral modification of Child's grading[13].

The following portal hemodynamic parameters were evaluated in the 87 patients by means of real-time ultrasound equipment with a 3,5-MHz-convex transducer with a pulse doppler device working at 3,5 and 2,5 MHz frequencies:

a) Diameter of the portal vein (PV) (mm) was calculated as the anteroposterior diameter during suspended respiration at the largest point.

b) Blood flow velocity of the portal vein (PV) (cm/min.) was calculated with the equipment from the doppler spectrum described by Gill[14]. The mean velocity ($V_{mean}$) of portal flow was measured. The sample volume, as large as at least half of the vessels caliber was positioned 1cm distal to the crossing point of the hepatic artery with the portal trunk in an oblique, subcostal scan.

c) Portal blood flow volume (PFV) (mm/min.) was calculated by the formula FVF=$V_{mean} \bullet \pi \bullet r^2$ where $r$ represents the diameter of the vessel.

d) Congestion index of the portal vein (CI): ratio of cross-sectional area and the blood flow velocity in the portal vein. The CI was calculated according to a modified Moriyasus's formula (15) using the $V_{mean}$ instead of the maximal flow velocity.

$$CI = \frac{(r)^2 \bullet \pi/10}{V_{mean}}$$

where $r$ is the vessels radius. For example, a portal vein diameter of 16mm and a portal flow velocity of 12 cm/min. yields a CI of 1.67.

In additon, the group of Siringo searched for spontaneous portal-systemic shunts and portal vein thrombosis in all patients. These two variables were referred to as morphological ultrasonographic findings.

The diagnosis of variceal hemorrhage was made when after an episode of hematemesis, melena or both emergency endoscopy (within 72 hours of the clinical manifestation of bleeding or index bleed), showed (a) active bleeding, a clot or a "white nipple" on a varix; (b) no lesions potentially responsible for bleeding when varices lacked the previously described signs; or (c) potential sources of hemorrhage other than varices but without signs of active or recent bleeding. Patients who did not undergo emergency endoscopy were considered to have bled from an unknown source. In this case the varices were considered the source of bleeding.

Thereafter, an univariate and multivariate analysis and a development of a prognostic model was performed including the mentioned risk factors. In this discriminant analysis the prognostic index, cut off points were found to identify patients who bled within 6 months (prognostic group I), after 6 six months (prognostic group II) or remained free of bleeding (prognostic group III). The cumulative proportion of patients correctly classified was 73% in the prognostic group I, 47% in the prognostic group II, and more than 80% in the prognostic group III. The addition of doppler ultrasound flowmetry to clinical, biochemical and endoscopic parameters only improves the classification of patients with early bleeding.

Thus, the cumulative rate of bleeding within 6 months in the group with early bleeding, as predicted by clinical and endoscopic criteria, was only 54%, quite poor and similar to 58%, as predicted by the North Italian Endoscopy Index of more than 40%[2]. The cumulative rate of actual bleeding predicted in this group was significantly improved (19% more) by the Congestion Index. However, the identification of patients with late occurrence of bleeding (after 6 months from entry) was poor in both models, with or without the inclusion of the Congestion Index (45 vs. 50%).

In conclusion, the study of Siringo *et al.*[5] shows, that a subgroup of cirrhotic patients is at a high risk of bleeding within 6 months of entry into the study. This subgroup of patients is best identified by a prognostic model based on clinical, endoscopic and doppler parameters. Patients who bled after 6 months from entry are poorly identifiable when only the status at entry is analyzed. Thus in this group the Congestion Index only poorly improved the bleeding risk criteria introduced by the North Italian Endoscopy Club (2). However, the criteria described by our group[9] are more reliable.

### References

[1] Burroughs, A.K., D'Heygere, F. and McIntyre, N. (1986). Pitfalls in studies of prophylactic therapy for variceal bleeding in cirrhotis. *Hepatology* **6**, 1407–1413

[2] North Italian Endoscopic Club for the Study and Treatment of Esophageal Varices:(1988). Prediction of the first hemorrhage in patient with cirrhosis of the liver and esophageal varices. *N. Engl. J. Med.* **319**, 983–989.

[3] Piai, G., Minieri, M., Catalano, M., D'Angelo, V., Sauro, A., Sabbatini, F. and Mazzacca, G. (1991). A prospective validation of two indexes of first variceal bleeding in liver cirrhosis (Abstract). *Gastroenterology* **100**, A784.

[4] Kleber, G., Sauerbruch, T., Ansari, H. and Paumgartner, G. (1991). Prediction of variceal hemorrhage in cirrhosis: a prospective follow-up study. *Gastroenterology* **100**, 1332–1337.

[5] Siringo, S., Bolondi, L., Gaiani, S., Sofia, S., Zironi, G., Rigamonti, A., DiFebo, G., Miglioli, M., Cavalli, G. and Barbaram, L. (1994). Timing of the first variceal hemorrhage in cirrhotic patients: prospective evaluation of doppler flowmetry, endoscopy and clinical parameters. *Hepatology* **2**, 66–73.

[6] Paquet, K.-J. (1982). Prophylactic endoscopic sclerosing treatment of the esophageal wall in varices – a prospective controlled randomized trial. *Endoscopy* **14**, 4–5.

[7] Japanese Research Society for Portal Hypertension. (1980). The general rules for recording endoscopic findings on esophageal varices. *Jpn. J. Surg.* **10**, 84–87

[8] Garcia-Tsao, G., Groszmann, R., Fisher, R., Conn, H., Atterbury, C. and Glickman, M. (1985). Portal pressure, presence of gastroesophageal varices and variceal bleeding. *Hepatology* **5**, 419–424.

[9] Paquet, K.-J., Kalk, J.-F., Klein, C.-P. and Gad, H.A. (1994). Prophylactic sclerotherapy for esophageal varices in high-risk cirrhotic patients selected by endoscopic and hemodynamic criteria: a randomized, single-center controlled trial. *Endoscopy* **26**, 734–740.

[10] Bolondi, L., Gaiani, S. and Barbara, L. (1991). Doppler flowmetry: clinical applications in portal hypertensive patients. In: Okuda K., Benhamou J-P, eds. Portal hypertension. Springer-Verlag, Tokyo, 161–182.

[11] Siringo, S., Di Febo, G., Bolondi , L., Gaiani , S., Vacirca, M., Miglioli, M. and Barbara, L. (1990). Predicting variceal bleeding in cirrhotics using endoscopy, hemodynamic evaluation by doppler ultrasound (DUS) and clinical variables (Abstract) *Gut* **31**. A1209.

[12] Gaiani, S., Bolondi, L., LiBassi, S., Zironi, G., Siringo, S. and Barbara L. (1991). Prevalence of spontaneous hepatofugal portal flow in liver cirrhosis: Clinical and endoscopic correlation in 228 patients. *Gastroenterology* **100**, 160–167.

[13] Cambell, D., Parker, D. and Anagnostopulos, C. (1973). Survival prediction in portacaval shunts: a computerized statistical analysis. *Am J. Surg* **126**, 748–751

[14] Gill, R. (1979). Pulsed doppler with B-mode imaging for quantitive blood flow measurement. *Ultrasound Med. Biol.* **5**, 223–235.

[15] Moriyasu, F., Nishida, O., Ban, N., Nakamura, T., Sakai, M., Miyake, T. and Uchino, H. (1986). "Congestion Index" of the portel vein. A J R (*Am J Roentgenol*) **146**, 735–739.

K-J Paquet, MD
Professor of Surgery
Lessingstrasse 8
D-97688 Bad Kissingen
Germany

### Liver and Pancreatic Resection in the Elderly

### ABSTRACT

*Fong, Y., Blumgart, L.H., Fortner, J.G and Brennan, M.F (1995). Pancreatic or liver resection for malignancy is safe and effective for the elderly. Annals of Surgery, 222: 426–437.*

*Background:* **Liver resection, or pancreaticoduodenectomy, has traditionally been thought to have a high morbidity and mortality rate among the elderly. Recent improvements in surgical and anesthetic techniques, an increasing number of elderly patients, and an increasing need to justify use of limited health care resources prompted an assessment of recent surgical outcomes.**

*Methods:* **Five hundred seventy-seven liver resections (July 1985–July 1994) performed for metastatic colorectal cancer and 488 pancreatic resections (October 1983–July 1994) performed for pancreatic malignancies were identified in departmental data bases. Outcomes of patients younger than age 70 years were compared with those of patients age 70 years or older.**

*Results:* **Liver resection for 128 patients age 70 years or older resulted in a 4% perioperative mortality rate and a 42% complication rate. Median hospital stay was 13 days, and 8% of the patients required admission to the intensive care unit (ICU). Median survival was 40 months, and the 5-year survival rate was 35%. No difference were found between results for the elderly and those for younger patients who had undergone liver resection, except for a minimally shorter hospital stay for the younger patients (median, 12 days *vs.* 13 days p=0.003). Pancreatic resection for 138 elderly patients resulted in a mortality rate of 6% and a complication rate of 45%. Median stay was 20 days, and 19% of the patients required ICU admission, results identical to those for the younger cohort. Long-term survival was poorer for the elderly patients, with a 5-year survival rate of 21% compared with 29% for the younger cohort (p=0.03).**

*Conclusions:* **Major liver or pancreatic resections can be performed for the elderly with acceptable morbidity and mortality rates and possible long-term survival. Chronologic age alone is not a contraindication to liver or pancreatic resection for malignancy.**

*Keywords*: Liver resection, hepatic resection, pancreatic resection, elderly major surgery

### PAPER DISCUSION

In recent years, advances in surgical practice have seen a reduction in morbidity and mortality rates for major hepatobiliary and pancreatic resectional surgery. Given that surgical resection is the only potential curative therapy for malignant disease of the liver and pancreas, many surgeons have advocated a more aggressive approach to the management of such malignancy in the anticipation of demonstrating improved long-term survival. Whilst the title of this paper from the Memorial Sloan-Kettering Cancer Center might support a relaxation of previously stringent selection criteria, the results require close scrutiny before other surgeons rush to follow suit and increase their own resectional practice.

In the management of pancreatic cancer, several recent series testify to the fact that pancreaticoduodenectomy can be undertaken with minimal mortality and low morbidity rates [1,2]. The authors report a creditable 6% mortality rate amongst the 138 patients over the age of 70 years undergoing pancreatic resection. The operative mortality rate was little different than for those patients less than 70 years of age undergoing similar resections. Five year survival was 21%, although none of the 10 patients surviving to five years underwent resection for pancreatic adenocarcinoma. The careful selection of patients for consideration of resection is exemplified by the fact that only 69 of the 138 patients undergoing pancreatic resection had pancreatic adenocarcinoma. The intense demands placed on hospital resource with such surgery is highlighted by the 45% complication rate and the 19% intensive care admission rate, although median hospital stay was 20 days. There are no data available to indicate whether recovery and return to normal activity was different in patients over 70 years of age and, as in similarly reported series, there is no assessment of the quality of life in patients following discharge.

The authors do recognise the potential effect of the pre-referral selection process in contributing to their good results. Further, it is noted that the authors have adopted the use of laparoscopy as a means of avoiding unnecessary laparotomy [3]. Our own experience suggests that a combination of laparoscopy and laparoscopic ultrasonography will avoid unnecessary non-therapeutic laparotomy [4], the morbidity of which is not often appreciated from the publication of selected patients undergoing resectional surgery. The median survival of 18 months following pancreatic resection reported by the authors merely serves to underline the importance of selecting out patients unlikely to benefit from an aggressive surgical approach.

A more encouraging role for hepatic resection in the management of colorectal metastases is evident from a number of studies which have demonstrated five year survival rates of up to 40% [5,6]. The present paper reports an encouraging 35% five year survival rate which is not dissimilar to the 39% five year survival rate observed in patients under the age of 70 years. Forty two percent of patients developed post-operative complications and there was a 4% peri-operative mortality rate. Male patients had a greater risk for complication than female patients and perhaps not surprisingly resection of at least one lobe of the liver and operative times exceeding four hours were associated with increased risk. It is unfortunate that such important factors of post-operative outcome may not therefore be easily predicted before patients are submitted to laparotomy. It is not readily evident from other reported series as to whether patients with other forms of hepatic malignancy can be similarly considered for resectional surgery with advancing years. Operative mortality rates as high as 41% have been reported over the last ten years for patients undergoing resection for primary hepatic malignancy [7,8,9].

It is evident that the authors are indeed able to conclude that "patients with pancreatic or liver malignancy should be considered for surgical therapy regardless of chronologic age". Nonetheless, it is apparent that assessment of individual risk for patients undergoing resectional surgery is not always possible. Relaxation of existing criteria for considering patients for complex hepatobiliary and pancreatic resection should therefore only be undertaken in established centres with a proven track record in this specialist field of surgery.

### *References*

[1] Trede, M., Schwall, G. and Saeger, H. (1990). Survival after pancreaticoduodenectomy. 118 consecutive resections without an operative mortality. *Annals of Surgery*, **211**, 447–458.

[2] Cameron, J.L., Pitt, H.A., Yeo, C.J., Lillemoe, K.D., Kaufman, H.S. and Coleman, J. (1993). One hundred and forty-five consecutive pancreaticoduodenectomies without mortality. *Annals of Surgery*, **217**, 430–435.

[3] Conlon, K.C., Dougherty, E., Kumstra, D.S., Coit, D.G., Turnbull, A.D.M. and Brennan, M.F. (1996). The value of minimal access surgery in the staging of patients with potentially resectable peripancreatic malignancy. *Annals of Surgery*, **223**, 134–140.

[4] John, T.G., Greig, J.D., Carter, D.C. and Garden, O.J. (1995). Carcinoma of the pancreatic head and periampullary region: tumour staging with laparoscopy and laparoscopic ultrasonography. *Annals of Surgery*, **221**, 156–164.

[5] Scheele, J., Stangl, R., Altendorf-Hofmann, A. and Paul, M. (1995). Resection of colorectal liver metastases. *World Journal of Surgery*, **19**, 59–71.
[6] Doci, R., Gennari, L., Bignami, P., Montalto, F., Morabito, A. and Bozzetti, F. (1991). One hundred patients with hepatic metastases from colorectal cancer treated by resection: analysis of prognostic determinants. *British Journal of Surgery*, **78**, 797–801.
[7] Yanaga, K., Kanematsu, T. and Takenaka, K. *et al.* (1988). Hepatic resection for hepatocellular carcinoma in elderly patients. *American Journal of Surgery*, **155**, 238–241.
[8] Fortner, J.G. and Lincer, R.M. (1990). Hepatic resection in the elderly. *Annals of Surgery*, **211**, 141–145.
[9] Nagasue, N., Chang, Y.C. and Takemoto, Y. *et al.* (1993). Liver resection in the aged (seventy years or older) with hepatocellular carcinoma. *Surgery*, **113**, 148–154.

O. J. Garden, MD, FRCS
Senior Lecturer in Surgery
Director of Organ Transplantation
University Department of Surgery
Royal Infirmary, Lauriston Place
Edinburgh
United Kingdom

### Pre-Liver Transplant: Tips Versus Distal Splenorenal Shunt

### ABSTRACT

*Abouljoud, M.S., Levy, M.F., Rees, C.R., Diamond, N.G., Lee, S.P., Mulligan, D.C., Goldstein, R.M., Husberg, B., Gonwa, T.A. and Klintmalm, G.B. (1995)A comparison of treatment with transjugular intrahepatic portosystemic shunt or distal splenorenal shunt in the management of variceal bleeding prior to liver transplantation. Transplantation, 59: 226–229.*

**Recurrent variceal bleeding in liver transplant candidates with end-stage liver disease can complicate or even prohibit a subsequent transplant procedure (OLT). Endoscopic sclero-therapy and medical therapy are considered as first-line management with surgical shunts reserved for refractory situations. Surgical shunts can be associated with a high mortality in this population and may complicate subsequent OLT. The transjugular intrahepatic portosystemic shunt (TIPS) has been recommended in these patients as a bridge to OLT. This is a new modality that has not been compared with previously established therapies such as the distal splenorenal shunt (DSRS). In this study we report our experience with 35 liver transplant recipients who had a previous TIPS (18 patients) or DSRS (17 patients) for variceal bleeding. The TIPS group had a significantly larger proportion of critically ill and Child-Pugh C patients. Mean operating time was more prolonged in the DSRS group ($P$=0.014) but transfusion requirements were similar. Intraoperative portal vein blood flow measurements averaged 2132±725 ml/min in the TIPS group compared with 1120±351ml/min in the DSRS group ($P$<0.001). Arterial flows were similar. Mean ICU and hospital stays were similar. There were 3 hospital mortalities in the DSRS group and none in the TIPS group ($P$=0.1). We conclude that TIPS is a valuable tool in the management of recurrent variceal bleeding prior to liver transplantation. Intraoperative hemodynamic measurements suggest a theoretical advantage with TIPS. In a group of patients with advanced liver disease we report an outcome that is similar to patients treated with DSRS prior to liver transplantation. The role of TIPS in the treatment of nontransplant candidates remains to be clarified.**

*Keywords*: TIPS, distal spenorenal shunt, liver transplant

### PAPER DISCUSSION

The paper by Abouljoud, *et al.* describes their experience with the use of transjugular intrahepatic portosystemic shunt (TIPS) in a busy transplant service and compares distal splenorenal shunts (DSRS) to TIPS relative to post-transplant outcome. Variceal bleeding in patients with portal hypertension is a frequent finding in patients with end stage liver disease [1–4]. It is critically important to assess risk factors for bleeding and to use preventive strategies in the evaluation and management of patients with known varices in the patient awaiting transplantation since variceal bleeding may exclude or complicate liver transplantation. Therapeutic options for treatment of bleeding esophagogastric varices and prevention of rebleeding should be structured to optimize patient outcome and assure successful transplantation.

Risk factors for predicting hemorrhage from varices have been well defined. Variceal size, intravariceal pressure, red color signs and the Child Pugh classification of liver disease are important variables in the evaluation of patients at risk for hemorrhage [5]. Lebrec *et al.* have shown that a portal or hepatic venous pressure gradient of at least 12 mm Hg is required for the developement of hemorrhage from esophageal varices, but that gradations in pressure above 12 mm Hg are not associated with a proportional increase in bleeding risk [6].

Prevention of variceal bleeding is important because mortality dramatically increases after bleeding occurs. Non-selective beta blockers may be used to prevent the first variceal hemorrhage in patients with end stage liver disease; patients with decompensated liver disease and medium to large varices are most likely to benefit [7,8]. Prevention of initial variceal hemorrhage through shunt surgery is not beneficial. Prophylatic sclerotherapy of varices is also not an effective preventive strategy [8,9]. Patients with end stage liver disease who bleed from esophageal varices warrant aggressive treatment to control the initial bleed and to prevent rebleeding. Acute bleeding may be controlled endoscopically with either sclerotherapy or band ligation [2–4,10]. Endoscopic ligation of varices controls acute bleeding as effectively as sclerotherapy, but with fewer complications, reduced rebleeding rates, and possibly improved survival [10]. Well-known complications associated with endoscopic treatment include esophageal ulceration or perforation, cardiopulmonary sequelae, and infections, all of which may preclude transplantation or increase operative risk [10–12]. Additionally, vasoactive agents may be used for the acute treatment of bleeding varices [13]. Vasopressin with nitroglycerin, glypressin, somatostatin, and octreotide may effectively control variceal hemorrhage, but only glypressin has been shown to significantly improve survival. In the patient who has recovered from a variceal bleed, the addition of non-selective beta blockers to endoscopic therapy reduces rebleeding rates when compared to either therapy alone but mortality rates may not be significantly improved [14,15].

What options are available for patients who continue to experience variceal bleeding despite sclerotherapy, variceal ligation and pharmacologic intervention? Both DSRS and TIPS control variceal hemorrhage and prevent rebleeding in over 90% of cases. As with all therapies, risks and benefits must be carefully considered. Rebleeding rates and patient survival are not significantly different between selective and non-selective shunts but the incidence of hepatic encephalopathy may be less in patients receiving DSRS [16–18].

TIPS controls acute variceal bleeding and prevents rebleeding in patients refractory to standard medical and endoscopic therapy. [19–22] TIPS has been associated with a variety of complications [20–22]. Fifteen to 66% of patients may develop shunt stenosis or occlusion and recurrent variceal bleeding within 1 year following TIPS placement. Seven to 30% of patients may experience new or worsening encephalopathy. In addition, improperly placed TIPS may also increase the difficulty of the transplant operation [23]. Potential complications resulting from TIPS should be carefully considered, keeping in mind the effect that complications may have on candidacy for liver transplant. TIPS is a short-term solution for the prevention of recurrent hemorrhage in patients with end stage liver disease who are candidates for transplantation.

What factors are important to consider in management of the potential transplant candidate with variceal hemorrhage? Several factors including the clinical status of the patient, the etiology and severity of liver disease, and candidacy for liver transplantation should be assessed before shunting or placement of TIPS. The etiology of liver disease influences survival of the patient with variceal bleeding. Patients with cholestatic liver disease tolerate variceal bleeding better than those with parenchymal liver disease [24, 25]. Severity of liver disease is important in determining whether to use DSRS or TIPS. Survival in patients with compensated liver disease receiving DSRS for variceal bleeding is superior to that of patients with decompensated liver disease [26]. Survival in patients who are treated with DSRS in the setting of mild to moderate liver disease is comparable to that of patients receiving allografts for a similar degree of liver failure, but patients with decompensated liver disease benefit more from transplantation [27, 28]. Surgical shunting should be reserved for patients with compensated liver disease and hemorrhage refractory to nonsurgical methods. TIPS followed by timely transplantation should be considered for patients with refractory variceal bleeding in the setting of decompensated liver disease [19, 20].

While transplantation is an effective therapy for end-stage liver disease; management of variceal hemorrhage needs to be carefully individualized to optimize patient outcome after transplantation. Abouljoud *et al.* describe their data comparing TIPS and DSRS in regards to safety, efficacy, long-term complications and influence on subsequent liver transplantation. Outcome parameters included operating time, number of transfusions, intraoperative portal venous and hepatic arterial flow measurements; length of stay and operative mortality was also assessed. Mean operating time was significantly longer in the DSRS group but there was no significant difference in transfusion requirements or cold ischemia times between the groups. Portal venous flow was significantly reduced in patients receiving DSRS compared to patients receiving TIPS but there was no significant difference in hepatic arterial flow between the groups. Analysis of the data demonstrated that patients undergoing TIPS has a similar post-operative course in terms of mortality, ICU and hospital stay compared to those patients who had DSRS even though they had significantly worse liver disease. Although not statistically significant, it is worth noting that 3 deaths occurred in the DSRS population and no deaths occurred in the TIPS group. The authors conclude that TIPS is a valuable tool in the management of patients with severe end stage liver disease awaiting transplantation who present with variceal hemorrhage.

A number of questions regarding methodology are raised in reviewing the paper by Abouljoud and colleagues that if clarified would be helpful in further defining the role of DSRS vs TIPS as adjunctive therapy in the potential transplant patient. The definition of refractory variceal hemorrhage was not provided. The authors state that follow-up ranged from 1–96 months. It would be helpful to know the duration of follow-up prior to and after liver transplantation. Recent studies suggest that 30 day post-TIPS survival in the non-transplant candidate with Child-Pugh class C liver disease and variceal bleeding is considerably worse when compared to that in patients with milder liver disease [29,30]. TIPS followed by timely transplantation in this patient population reduces mortality rates; this may account for the promising results in patients with Child-Pugh class C liver disease receiving TIPS.

What practical conclusions can we draw from Abjoulboud's experience? Their data would suggest that the use of TIPS can safely serve as a bridge to transplantation and is particularly useful in the patient with advanced liver disease. Although the numbers were too small to draw firm statistical conclusions, there were three hospital mortalities in the DSRS group and none in the TIPS group even though there was a larger proportion of critically ill and Child-Pugh class C patients in the TIPS group. However, the role of DSRS in the treatment of variceal hemorrhage should not be minimized. In the patient with relatively well preserved synthetic function and recurrent variceal hemorrhage, DSRS is a time-tested, proven and effective treatment. The application of a less-invasive treatment modality in the hemorrhaging liver patient adds another alternative to our arsenal of therapeutic options. As is the case in all studies of new treatment modalities, the study suffers from small numbers in each group. While the results of Abouljoud and colleagues are encouraging a larger, prospective, randomized, controlled trial comparing endpoints of efficacy and safety of DSRS and TIPS would be helpful in defining standards of practice in the patient awaiting transplantation.

### References

[1] Navarro, V.J. and Garcia-Tsao, G. (1995). Varicealhemorrhage. *Critical Care Clinics*, **11**, 391–414.

[2] Terblanche, J., Burroughs, A.K. and Hobbs, K.E. (1989). Controversies in the management of bleeding esophageal varices. *New England Journal of Medicine*, **320**, 1393–1398, 1469–1475.

[3] Lebrec, D. (1992). Portal hypertension in complications of Chronic Liver Disease, edited by Rector, W. pp. 24–67, St. Louis: Mosby-Year Book, Inc.

[4] Reyes, J., Irvatsuki, S. (1992). Current management of portal hypertension with liver transplantation. *Advances in Surgery*, **25**, 189–208.

[5] The North Italian Endoscopic Club for the Study and Treatment of Esophageal Varices (1988). Prediction of the first variceal hemorrhage in patients with cirrhosis of the liver and esophageal varices: A prospective multi-center study. *New England Journal of Medicine*, **319**, 983–989.

[6] Lebrec, D., Fleury, O., Reu, B., Nahum, H. and Benhamou, J.P. (1980). Portal hypertension, size of esophageal varices and risk of gastrointestinal bleeding in alcoholic cirrhosis. *Gastroenterology*, **79**, 1139–1144.

[7] Poynard, T., Cales, P., Pasta, L., Ideo, G., Pascal, J.P., Pagliaro, L. and Lebrec, D. (1991). Beta-adrenergic-antagonist drugs in the prevention of gastrointestinal bleeding in patients with cirrhosis and esophageal varices: an analysis of data and prognostic factors in 589 patients from four randomized clinical trials. *New England Journal of Medicine*, **32**, 1532–1538.

[8] Pagliaro, L., D'Amico, G., Sorenson, T.I.A., Lebrec, D., Burroughs, A.K., Morabito, A., Tine, F., Politi, F. and Traina, M. (1992). Prevention of first bleeding in cirrhosis: a meta-analysis of randomized trials of nonsurgical treatment. *Annals of Internal Medicine*, **117**, 59–70.

[9] Van Ruiswyk, J. and Byrd, J.C. (1992). Efficacy of sclerotherapy for prevention of a first variceal hemorrhage. *Gastroenterology*, **102**, 587–597.

[10] Stiegmann, G.V., Go, J.A., Michaltez-Onody, P.A., Korula, J., Lieberman, D., Saeed, Z.A. and Reveille, M.R. (1992). Endoscopic scletotherapy as compared with endoscopic ligation for bleeding esophageal varices. *New England Journal of Medicine*, **326**, 1527–1532.

[11] Health and Public Policy Committee (1984). Endoscopic sclerotherapy for esophageal varices. *Annals of Internal Medicine*, **100**, 608–610.

[12] Gimson, A.E., Ramage, J.K., Panos, M.Z., Hayllar, K., Harrison, P.M., Williams, R. and Westaby, D. (1993). Randomized trial of variceal banding ligation versus injection sclerotherapy for bleeding oesophageal varices. *The Lancet*, **342**, 391–394.

[13] D'Amico, G., Luigi, P. and Bosch, J. (1995). The treatment of portal hypertension: A meta-analytic review. *Hepatology*, **22**, 332–354.

[14] Jensen, L.S. and Krarup, N. (1989). Propranolol in the prevention of rebleeding from esophageal varices during the course of endoscopic sclerotherapy. *Scandinavian Journal of Gastroenterology*, **24**, 339–345.

[15] Ink, O., Martin, T., Poynard, T., Reville, M., Anciaux, M.L., Lenoir, C., Marill, J.L., Lebadie, H., Masliah, C. and Perrin, D. (1992). Does elective sclerotherapy improve the efficacy of long term propranolol for prevention of recurrent bleeding in patients with severe cirrhosis? A prospective multi-center randomized trial. *Hepatology*, **16**, 912–919.

[16] Rikkers, L., Rudman, D. and Galambos, J. (1978). A randomized controlled trial of distal splenorenal shunts. *Annals of Surgery*, **188**, 271–282.

[17] Reichle, F., Fahmy, W. and Golsorkhi, M. (1979). Propsective comparative clinical trial with distal splenorenal and mesocaval shunts. *American Journal of Surgery*, **137**, 13–21.

[18] Millikan, W.,Warren, W., Henderson, J., Smith, R.B. III, Salam, A.A., Galambos, J.T., Kutner, M.H. and Keen, J.H. (1985). The Emory prospective randomized trial: Selective versus nonselective shunt to control variceal bleeding. Ten year follow-up. *Annals of Surgery*, **201**, 712–722.

[19] Ring, E., Lake, J., Roberts, J., Gordon, R., LaBerge, J., Read, A., Sterneck, E. and Ascher, N. (1992). Using transjugular intrahepatic portosystemic shunts to control variceal bleeding before liver transplantation. *Annals of Internal Medicine*, **116**, 304–306.

[20] Rossle, M., Haag, K., Ochs, A., Sellinger, M., Noldge, G., Perarneu, J., Berger, E., Blum, V., Gabelmann, A. and Hauenstein, K. (1994). The trasjugular intrahepatic portosystemic stent shunt procedure for variceal bleeding. *New England Journal of Medicine*, **330**, 165–171.

[21] LaBerge, J.M., Ring, E., Gordon, R., Lake, J., Doherty, M., Somberg, K., Roberts, J. and Ascher, N. (1993). Creation of transjugular intrahepatic portosystemic shunts with the wallstent endoprosthesis: Results in 100 patients. *Radiology*, **187**, 413–420.

[22] Shiman, M., Jeers, L., Hoofnagle, J. and Tralka, T. (1995). The role of transjugular intrahepatic shunt (TIPS) for treatment of portal hypertension and its complications. National Digestive Diseases Advisory Board. *Hepatology*, **22**, 1591–1597.

[23] Millis, J.M., Martin, P., Gomes, A., Shaked, A., Coliquhoun, S.D., Jurim, O., Goldstein, L. and Busuttil, R.W. (1995). Transjugular intrahepatic portosystemic shunts: Impact on liver transplantation. Liver Transplantation and Surgery, **1**, 229–233.

[24] Gores, G.J., Wiesner, R.H., Dickson, E.R., Zinmeister, A.R., Jorgensen, R.A. and Langworthy, A. (1989). Prospective Evaluation of esophageal varices in primary biliary cirrhosis. *Gastroenterology*, **96**, 1552–1559.

[25] Graham, D.Y. and Smith, J.L. (1981). The course of patients after variceal hemorrhage. *Gastroenterology*, **80**, 800–809.

[27] Wood, P., Shaw, B. and Rikkers, L. (1990). Liver transplantation for variceal hemorrhage. Surgical Clinics of North America, **70**, 449–461.

[28] Bismuth, H., Adam, R., Mathur, S. and Sherlock, D. (1990). Options for elective treatment of portal hypertension in cirrhotic patients in the transplantation era. *American Journal of Surgery*, **160**, 105–110.

[29] Coldwell, D.M., Ring, E.J., Rees, C.R., Zemel, G., Darcy, M.D., Haskal, Z.J., McKusick, M.A. and Greenfield, A.J. (1995). Multi-center investigation of the role of transjugular intrahepatic portosystemic shunt in the management of portal hypertension. *Radiology*, **196**, 335–340.

[30] Rubin, R.A., Haskal, Z.J., O'Brien, C.B., Cope, C. and Brass, C.A. (1995). Transjugular intrahepatic portosystemic shunting: Decreased survival for patients with high APACHE II scores. *American Journal of Gastroenterology*, **90**, 556–563.

Thomas W. Faust M. D.
Michael F. Sorrell M. D.
University of Nebraska Medical Center
Liver Transplant Program

Michael F. Sorrell, M. D.
University of Nebraska Medical Center
Liver Transplant Program
600 S. 42nd Street
Omaha, Nebraska 68198–3280

### Is Hepatectomy Necessary in Dealing with Left Hepatolithiasis with Intrahepatic Duct Stricture?

### ABSTRACT

*Sheen-Chen, S-M., Cheng, Y-F., Chou, F-F.* and *Lee, T-Y. (1995). Ductal dilatation and stenting make routine hepatectomy unnecessary for left hepatolithiasis with intrahepatic biliary stricture. Surgery; 117: 32–36.*

*Background:* **Hepatolithiasis with intrahepatic biliary strictures, more common in Southeast Asia than elsewhere, remains a difficult problem to manage. Hepatic resection has recently been advocated as one of the treatment modalities for hepatolithiasis; however, this procedure is not without risk. This study was designed to achieve complete clearance of the stones, eliminate bile stasis, and avoid the potential risks of hepatic resection in the patient with hepatolithiasis and intrahepatic biliary stricture.**

*Methods:* **In this prospective clinical trial 13 patients with retained left hepatolithiasis and intrahepatic biliary strictures were included. All the patients met the following criteria: (1) initial surgical procedure for hepatolithiasis, (2) normal gross findings of the left liver, and**

